# Cardiovascular disease and the risk of incident falls and mortality among adults aged ≥ 65 years presenting to the emergency department: a cohort study from national registry data in Denmark

**DOI:** 10.1186/s12877-023-04618-2

**Published:** 2024-01-24

**Authors:** Aisling M. O’Halloran, Jolien Cremers, Karsten Vrangbæk, Lorna Roe, Robert Bourke, Laust H. Mortensen, Rudi G. J. Westendorp, Rose Anne Kenny

**Affiliations:** 1 Medical Gerontology, School of Medicine, Trinity College Dublin, Trinity Central, 152-160 Pearse Street, Dublin, Ireland; 2 Data Science Lab, Statistics Denmark, Copenhagen, Denmark; 3 Department of Public Health, University of Copenhagen, Copenhagen, Denmark; 4 Center for Healthy Aging, University of Copenhagen, Copenhagen, Denmark; 5 Centre for Health Economics and Policy, University of Copenhagen, Copenhagen, Denmark; 6 Department of Political Science, University of Copenhagen, Copenhagen, Denmark; 7 Global Brain Health Institute (GBHI), Trinity College Dublin, Dublin, Ireland; 8 Centre for Health Policy and Management, Trinity College Dublin, Dublin, Ireland; 9 Mercer’s Institute for Successful Ageing, St. James’s Hospital, Dublin, Ireland

**Keywords:** Falls, ED, Cardiovascular disease, Mortality

## Abstract

**Background:**

Falls cause 58% of injury-related Emergency Department (ED) attendances. Previous research has highlighted the potential role of cardiovascular risk factors for falls. This study investigated the impact of cardiovascular disease (CVD) risk on three-year incident falls, with presentation to the ED, and mortality.

**Methods:**

A matched cohort study design was employed using national registry data from 82,292 adults (33% male) aged ≥ 65 years living in Denmark who attended the ED in 2013. We compared age and gender matched ED attendees presenting with a fall versus another reason. The cohort was followed for three-year incident falls, with presentation to the ED, and mortality. The impact of falls-related CVDs was also examined.

**Results:**

Three-year incident falls was twofold higher among age and gender matched ED attendees aged ≥ 65 years presenting with a fall versus another reason at baseline. A presentation of falls with hip fracture had the highest percentage of incident falls in the 65–74 age group (22%) and the highest percentage mortality in all age groups (27–62%). CVD was not a significant factor in presenting with a fall at the ED, nor did it contribute significantly to the prediction of three-year incident falls. CVD was strongly associated with mortality risk among the ED fall group (RR = 1.81, 95% CI: 1.67–1.97) and showed interactions with both age and fall history.

**Conclusion:**

In this large study of adults aged ≥ 65 years attending the ED utilising data from national administrative registers in Denmark, we confirm that older adults attending the ED with a fall, including those with hip fracture, were at greatest risk for future falls. While CVD did not predict incident falls, it increased the risk of mortality in the three-year follow up with advancing age. This may be informative for the provision of care pathways for older adults attending the ED due to a fall.

**Supplementary Information:**

The online version contains supplementary material available at 10.1186/s12877-023-04618-2.

## Introduction

Falls are an evolving frailty state and a major public health challenge among older adults [1]. One-third of people over the age of 65 fall each year, increasing to 50% by the age of 80 years [2, 3]. Of those who have fallen, half will experience future falls [4, 5]. In 2015 within the EU-28, there were an estimated 35,848 fall related deaths each year amongst people aged ≥ 65 years [6]. However, this is likely an underestimation. Falls account for 58% of injury-related ED attendances. By 2050, it is projected that the annual number of fall-related ED attendances in the EU will increase to over 6 million with an annual expenditure exceeding 45 billion euros [6]. Therefore, it is important to identify modifiable factors that predict falls, thereby improving falls prevention and reducing expenditures.

Falls result from the interaction of multiple and diverse intrinsic, behavioural and extrinsic risk factors, some of which can be modified [7, 8]. Having a previous fall and living with frailty are both strong predictors of future falls [9, 10]. Medication types and polypharmacy (taking 5 + medications concurrently) are modifiable extrinsic risk factors that also predict falls and recurrent falls [11, 12].

Whereas some studies report decreases in fall rates, after targeted intervention, of 25% to 40%, [13, 14] a recent systematic review and meta-analysis found more modest reductions for falls (12–30%) and recurrent falls (0–22%) [15]. The recent World Falls Prevention Guidelines underline the significance of cardiovascular assessment (CVD history and autonomic function testing) and management for falls prevention [16]. However, the evidence that drug and musculoskeletal interventions for CVD reduce the causes of falls is comparatively sparse and comprehensive cardiovascular assessment is not routine practice for all individuals who fall [17, 18], including in the busy ED environment. ED attendance and admission rates increase significantly from age 65–69 up to 90 + years, largely driven by acute but also non-specific medical diagnoses [19, 20]. In the context of the ED, expedient decisions regarding triage must be made and rely on risk stratification.

Cardiovascular diseases (CVDs) may contribute to a fall by inducing cerebral hypoperfusion, resulting in transient imbalance, dizziness, or temporary loss of consciousness [21]. Our group have previously published two systematic reviews and meta-analyses of 86 and 156 studies respectively, which showed strong associations between CVDs and falls with the most consistent associations observed for hypertension, orthostatic hypotension (OH), arrhythmias, carotid sinus hypersensitivity, vasovagal syncope, heart failure and cardiac arrhythmia [22, 23].

Given our recent work highlighting the association between particular CVDs and falls, we employed one of the largest datasets from national administrative registers in Denmark to determine the risk of incident falls presenting to the ED and mortality over three years among ED attendees aged ≥ 65 years. We examined whether CVDs known to be associated with falls, were associated with and predictive of incident falls and mortality among fallers presenting to the ED.

## Methods

### Data sources

This study involved the secondary analyses of data from census based administrative registers in Denmark (https://www.dataforgood.science) under the central authority of Statistics Denmark (https://www.dst.dk/en#). This is a protected data environment and one of the world's most extensive data repositories. It maintains hundreds of high quality registers and produces fine-grained statistics on changes in many aspects of life e.g. social, economic, biomedical conditions, and geographical location over time [24].

In this study, data on demographics, prescription medications, and hospital administrative and clinical information were analysed from the following registries respectively: The Danish Civil Registration System; The National Prescription Registry; and The Danish National Patient Registry [25–27]. Mortality data was obtained from the Death Register, a fully digitalized register including all deaths of Danish residents dying only in Denmark [28].

### Study population

An overview of the study design is provided in Supplementary Appendix 1. From the cohort of all individuals aged 65 and older presenting at the ED for any cause in 2013 (*n* = 135,655), we selected individuals presenting at the ED with a fall and an age and sex matched sample of individual presenting to the ED for a reason other than a fall (*n* = 41,146 ED fallers and *n* = 41,146 age and sex matched non-faller ED attendees). Common reasons for older adults visiting the ED for reasons other than a fall include ischemic heart disease, congestive heart failure, syncope, cardiac dysrhythmias, acute cerebrovascular accidents, pneumonia, abdominal disorders and urinary tract infections, This approach was taken to compare ‘faller’ to ‘non-faller’ ED attendees. Every individual in the study population was followed from the index date (admission to the ED in 2013) until death, emigration, or three years after the index date. Thirty-seven (0.09%) of fallers could not be followed for three years due to emigration. Median follow up times and interquartile ranges (IQR) for different age groups are provided (Supplementary Appendix 2).

### Demographic variables

*Age and sex:* These variables were obtained from The Danish Civil Registration System which contains all persons alive and living in Denmark. It was updated annually every December until 2007 and every three months since 2008. For this study, we accessed data on individuals aged 65 and over. Data was classified by sex and three age categories: 65–74; 75–84 and 85 + years.

### Medication variables

We used information on the purchase date and the Anatomical Therapeutic Chemical (ATC) classification system codes of prescription medications [29] redeemed by Danish residents at community pharmacies, including drugs prescribed to nursing home residents, from The National Prescription Registry. Medications were classified by distinct substances according to the fifth level of the ATC classification system. ATC codes were obtained from the National Prescription registry for the three years prior to the index fall.

### Cardiovascular medications

This variable included those medications with level 1 ATC code “C” (cardiovascular system).

### Polypharmacy

This was defined as having a yearly intake of at least 5 medications and excessive polypharmacy as a yearly intake of at least 10 medications. Thus, three distinct groups were defined: no polypharmacy (< 5 medications), polypharmacy (5 + medications) and excessive polypharmacy (10 + medications).

### Hospital administrative and clinical variables

In our analysis we used information from the Danish National Patient Registry (DNPR), which details administrative and clinical data on all patient contacts with Danish non-psychiatric hospitals since 1977 and psychiatric specialty clinics or hospitals since 1995 [30].

*Falls were* defined as ED visits with any of the following registration International Classification of Diseases-10 (ICD-10) based codes: EUHE00-03, EUHE08-EUHE09 (2008–2013) and EUBA-EUBB (2014–2015).

### Index fall

The index fall was defined as the first contact in the DNPR at the ED because of a fall in the year 2013.

### Post-index fall

This was defined as a fall in the DNPR that occurred in the period from the discharge day of the index fall up to and including three years after the admission date of the index fall.

### Incident falls

An individual was classified as having an incident fall if he/she had at least one post-index fall *(*≥ *1 versus 0 post-index falls)*, with presentation to the ED, within three years after the ED presentation date of the index fall in 2013, or the ED presentation date in the case of the non-fallers.

### Pre-index falls

This was defined as falls in the DNPR with an admission date up to three years before the ED presentation date of the index fall in 2013, or the ED presentation date in the case of the non-fallers.

### Falls history

An individual was classified as having a fall history if he/she has had at least one pre-index fall, during the three years prior to the admission date of the index fall.

### Cardiovascular diseases, diagnoses and medical history

Diagnoses of CVDs associated with falls risk, as evidenced from the literature, were identified by ICD10 codes in the DNPR. They included hypertension disease (I10-I15), ischaemic heart diseases (I20-I25), atrial fibrillation and flutter (I48), other cardiac arrhythmias (I49), heart failure (I50), cerebrovascular diseases (I60-I69) and hypotension (I95). An individual is said to have a diagnosis if he/she has received one of these CVD or heart rhythm disorder diagnosis within five years before the admission date of the index fall.

### Hip fractures

This was defined as hip fracture resulting from a fall and were identified as hip fracture diagnoses from the DNPR that were registered between the admission and discharge date of the index fall (ICD-10 codes S72.0 and S72.1).

### Hospitalizations

A hospitalization resulting from a fall was classified as hospitalization from the DNPR that lasted for more than 1 day and had the same admission date as the index fall.

### Mortality

Mortality incidence was computed for individuals that were deceased within 3 years of the surrogate or index fall in 2013 using data obtained from the Central Persons Register.

### Statistical analysis

The main outcomes of interest were incident falls (≥ 1 versus 0 post-index falls) and mortality over three years, following the index fall in 2013, or ED presentation date for non-fallers.

Descriptive characteristics for the demographic, medication and clinical indicators in the fall and matched non-fall groups from the cohort were calculated using means and standard deviations for continuous variables and proportions for categorical variables. This data is presented in Table 1.

**Table 1:** Descriptive characteristics of age and sex matched ED fall and non-fall groups

	(ED Fall)	(ED Non-fall)	*P*-value
N	41,146	41,146	-
Sex (Males)	0.33	0.33	1.00
Age^a^ (SD)	78.29(8.85)	78.20 (8.77)	0.14
Cardiovascular disease^b^^,c^	0.40	0.45	< 0.01
Hypertensive diseases	0.26	0.29	< 0.01
Ischaemic heart diseases	0.12	0.15	< 0.01
Atrial fibrillation and flutter	0.12	0.15	< 0.01
Other cardiac arrhythmias	0.02	0.03	< 0.01
Heart failure	0.06	0.08	< 0.01
Cerebrovascular diseases	0.11	0.11	1.00
Hypotension	0.02	0.02	1.00
Cardiovascular medication	0.78	0.42	0.07
Polypharmacy (5–10)	0.22	0.09	0.03
Excessive polypharmacy (> 10)	0.65	0.36	< 0.01
Fall history	0.20	0.14	< 0.01
Hip fractures	0.04	0.01	< 0.01
Hospitalisations	0.36	0.47	< 0.01

Data are presented as proportions unless indicated otherwise

^a^Mean (Standard Deviation)

^b^Proportion of CVD in ED Fall group for the analysis of New CVD in Appendices 3 and 4 was 0.47

^c^Proportion of CVD in ED Fall group for the sensitivity analysis in Appendices 7 and 8 was 0.40

**Fig. 1:**
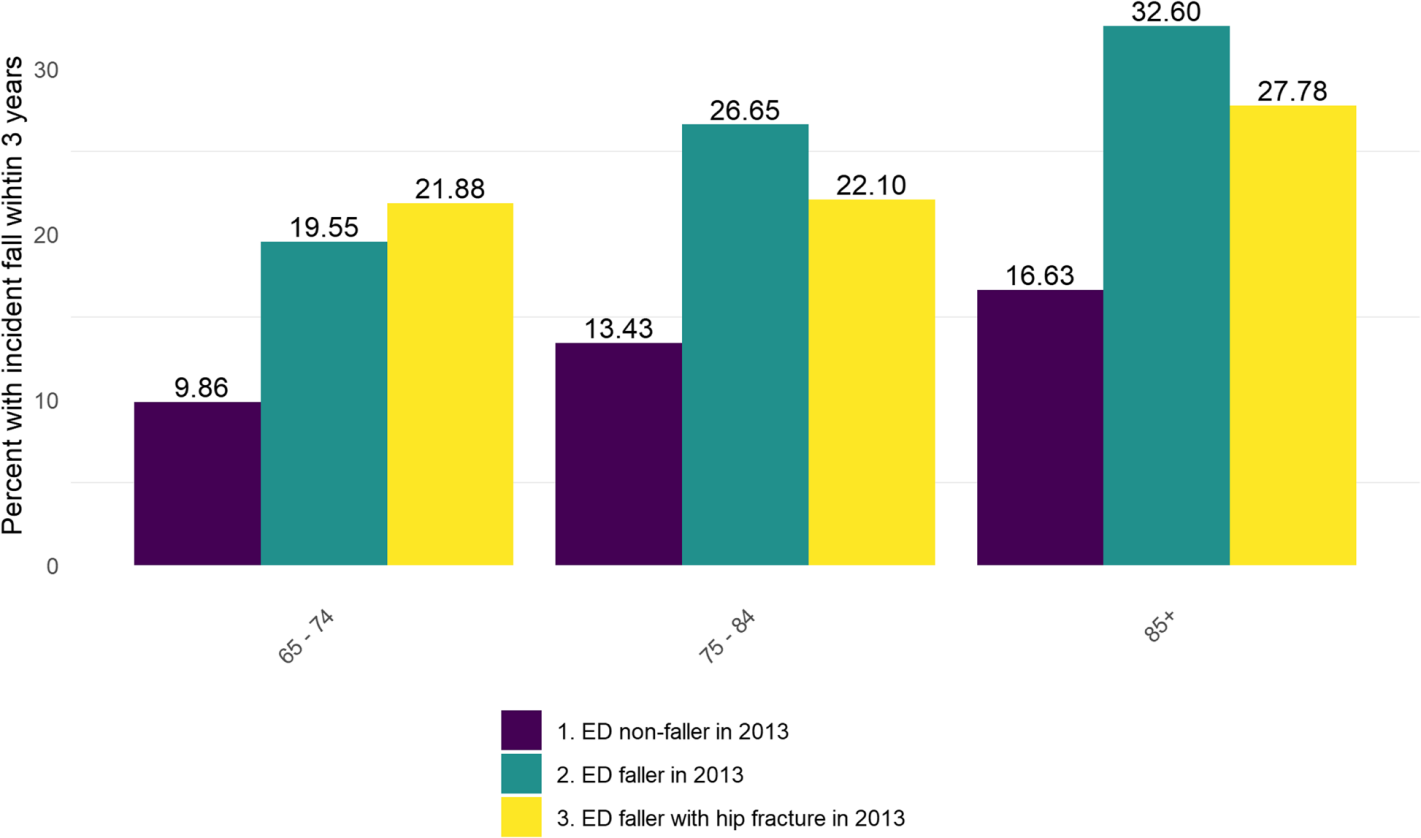
The absolute risk of incident falls (≥ 1) among the fall, fall with hip fracture and non-fall ED attendee groups by age

Incident falls, stratified by age groups (65–74, 75–84, 85 + years), was computed and plotted as proportions (range 0–1) for the non-fall, fall, and fall with hip fracture ED attendee groups. This data is presented in Fig. 1.

We investigated prospective associations between available clinical indicators at the time of the index fall and incident falls over three years by computing adjusted relative risks (RR) and corresponding 95% confidence intervals (CI). The adjusted risk ratios were estimated using log-binomial regression or regression with robust standard errors for those analyses in which the log-binomial model did not converge, as recommended by Knol and colleagues. [30] The clinical indicators investigated were age, hip fracture, falls history, polypharmacy, medical history of a diagnosed CVD, prescribed CVD medication and two-way interactions of age with CVD diagnosis, and falls history with CVD diagnosis. For second order interactions between CVD and age and CVD and fall history, we computed the RR per stratum, multiplicative interaction RR and Relative Excess Risk due to Interaction (RERI), as estimates of the additive interaction, as recommended by Knol and colleagues [28]. This data is presented in Table 2.

**Table 2:** Relative risk of three-year incident falls (≥ 1 versus 0) and all-cause mortality associated with clinical indicators within the ED fall group

	Incident falls^**a**^ (≥ 1 versus none)	All-cause mortality^**a**^
	(*n* = 41,146)	(*n* = 41,146)
Intercept	1.00	1.00
Age 75–84	1.32 (1.25; 1.40)*	2.32 (2.16; 2.49)*
Age 85 +	1.54 (1.45; 1.63)*	4.89 (4.58; 5.22)*
Hip fracture	0.88 (0.81; 0.95)*	1.31 (1.25; 1.38)*
Fall history (1 fall)^b^	1.50 (1.42; 1.59)*	1.12 (1.06; 1.18)*
Fall history (> 1 fall)^b^	2.04 (1.89; 2.19)*	1.41 (1.31; 1.53)*
Polypharmacy^b^	1.33 (1.24; 1.44)*	1.44 (1.32; 1.56)*
Excessive polypharmacy^b^	1.51 (1.40; 1.62)*	2.07 (1.91; 2.24)*
Cardiovascular medication use^b^	0.94 (0.89; 0.98)*	0.95 (0.91; 0.99)*
Cardiovascular disease^c^	1.33 (1.24; 1.43)*	1.81 (1.67; 1.97)*
Cardiovascular disease:Age 75–84	1.50 (1.41; 1.60)*	3.31 (3.05; 3.58)*
Cardiovascular disease:Age 85 +	1.67 (1.57; 1.77)*	5.35 (4.95; 5.77)*
Cardiovascular disease:Fall history (1 fall)	1.84 (1.70; 1.99)*	2.00 (1.80; 2.22)*
Cardiovascular disease:Fall history (> 1 fall)	2.37 (2.17; 2.58)*	2.32 (2.06; 2.62)*

^a^Relative risk (RR) with 95% confidence intervals (95% CI). The last four lines represent the RR of the combination of groups (having CVD and in a specific age group or having a specific fall history) compared to the reference 65–74 year olds without CVD or fall history. Note that these four lines do not represent a test of the interaction of the effects of CVD, age and fall history

^b^Three years prior to the index fall

^c^A diagnosed cardiovascular disease during the actual medical history (five years prior to the index fall)

^*^significance level p < 0.05

Data examining the additional impact on incident falls and mortality, of a new CVD diagnosis within 90 days of the index fall presentation to the ED is also provided in Appendices 3 and 4 with sensitivity analysis provided in Appendices 7 and 8.

**Fig. 2:**
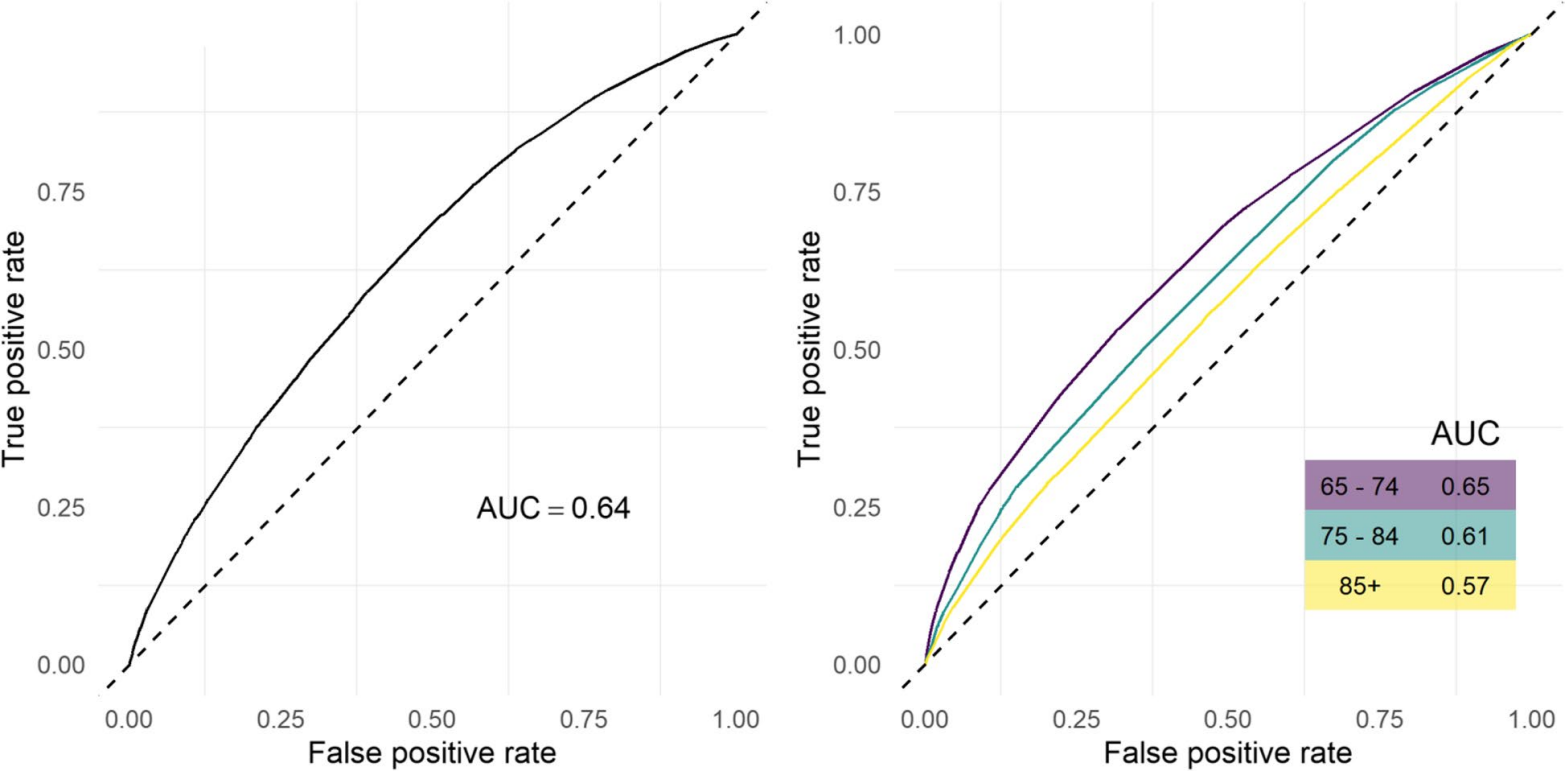
Receiver operating curves predicting incident falls (≥ 1 versus 0) over three years for the ED fall group aged ≥ 65 years (left) and per age group (right) together with the AUC, based on logistic regression models. The dotted line indicated an AUC of 0.5. Note that the logistic regression models for the ROCs include all second order interactions except those between polypharmacy and excessive polypharmacy, CVD and heart rhythm disorder diagnoses, and fall history (1 fall) and fall history (> 1 fall) due to singularity

Finally, we examined the predictive power of the clinical indicators from the above analyses to discriminate incident falls stratified by age groups (65–74, 75–84, 85 + years). This was performed by fitting a logistic regression model in which the clinical indicators were included as predictors and the binary incident falls indicator as the outcome. The logistic regression models included all second order interactions except those between polypharmacy and excessive polypharmacy and fall history (1 fall) and fall history (> 1 fall) due to singularity. Then we plotted the receiver operating curves (ROC) and computed the area under the curve (AUC) as a measure of discrimination. This data is presented in Fig. 2. In Appendix 5 we present a similar analysis in which the CVD diagnosis variable is excluded to investigate the additional predictive power that CVDs provide.

Mortality risk was computed as the proportion of individuals that were deceased within 3 years of the index fall or ED visit by age group. We also stratified our analysis by ED fall with a fracture (Appendix 6). Prospective associations between clinical indicators at the time of the index fall and mortality over three years were investigated by computing adjusted RR scores and corresponding 95% CI (Table 2, and Appendices 3 and 4). Similar to the analysis for incident falls risk, we examined the predictive power of the clinical indicators by a logistic regression model. We plotted the receiver operating curves (ROC) and computed the area under the curve (AUC) as a measure of discrimination. This data is presented in Appendix 9. We used R statistical software package version 3.6.1 to perform the analyses.

Statistical significance is indicated at the level of *p* ≤ 0.05.

## Results

### Characteristics of fall and non-fall groups of ED attendees

Supplementary Appendix 1 presents the study design utilised to compare ED attendees presenting with a fall (ED fall) to age and sex matched ED attendees presenting for a reason other than a fall (ED non-fall). ED fall and non-fall groups were matched by age (mean: 78.3 years) and sex (33% male) in Table 1. Compared to the ED non-fall group, the ED fall group exhibited more polypharmacy and excessive polypharmacy, with a higher proportion taking cardiovascular medications. However, the proportion with a previous diagnosis of a CVD (evidenced to be related to falls risk) was lower among the ED fall group. A higher proportion of the ED fall group had a history of falls and hip fracture. However, the proportion of individuals hospitalized for > 1 day at the time of the index ED attendance in 2013 was higher among the ED non-fall group.

### *Incident falls (*≥ *1) among fall versus non-fall groups of ED attendees*

Figure 1 presents the percentage of incident (post-index) falls over three years of follow up for ED attendees aged ≥ 65 years with no fall, a fall, and a fall with hip fracture, at the index ED admission in 2013. The data is presented for 65–74, 75–84 and 85 + age groups. Within each group of ED attendees, there was a progressive age-associated increase in the percentage with incident falls. Within each age group, the percentage of incident falls among the ED fall group; was twice that observed for the non-fall group. Only within the 65–74 age group was the percentage of incident falls highest among the fall with hip fracture group.

### *Clinical risk indicators for incident falls (*≥ *1 versus none) within the ED fall group*

Table 2 presents prospective associations – adjusted relative risk ratio (RR) with 95% confidence intervals (95% CI)—between clinical risk indicators at the index fall and incident falls over 3 years. Age (75–84 years: RR = 1.32, and 85 + years: RR = 1.54), a history of falls (1 fall: RR = 1.50, and > 1 fall: RR = 2.04), polypharmacy (5–10 medications: RR = 1.33, and > 10 medications: RR = 1.51) and CVD diagnosis (RR = 1.33) were statistically significant predictors of incident falls over 3 years within the ED fall group. Taking CVD medications (RR = 0.94) and hip fracture (RR = 0.88) reduced the risk of incident falls in fully adjusted models.

Compared to the ED fall group aged 65–74 years without a CVD diagnosis, having a CVD diagnosis and being aged 75–84 increased the adjusted RR of incident falls by 50%, this increased further to 67% for the ED fall groups with a CVD diagnosis in the 85 + age group. Similarly, a CVD diagnosis and one pre-index fall increased the risk of incident falls by 88% in the ED fall group, while a CVD diagnosis and > 1 pre-index fall increased the risk of incident falls more than twofold compared to having no CVD diagnosis with no pre-index falls.

To fully investigate the second order interactions between CVD and age, and CVD and fall history, we computed the RR per stratum, multiplicative interaction RR, and Relative Excess Risk due to Interaction (RERI) to estimate the additive interaction (Appendix 4) [30]. The RR per stratum shows the relative risk of incident falls within age groups and groups with pre-index falls. Compared to the ED fall group aged 65–74 years, being aged 75–84 increased the adjusted RR of incident falls by 13%, with a 33% increased risk in the 85 + age group. Compared to the ED fall group without a pre-index fall, having one pre-index fall increased the adjusted RR of incident falls by 23%, with a 16% increased risk for > 1 pre-index fall. The multiplicative interaction effects between CVD, age and fall history show that the combined effect of having a CVD diagnosis and being older is less than the product of the individual effects of CVD and age (RR = 0.85 for the 75–84 group and RR = 0.81 for the 85 + age group). This means that having a CVD diagnosis has a protective effect on the incident fall risk for the older ED fall group on a multiplicative scale. Having a CVD diagnosis also has a protective effect on the incident fall risk for those with > 1 pre-index fall (RR = 0.87). The RERI or additive interaction effect shows that the combined effect of having a CVD diagnosis and being older is less than the sum of the individual effects of CVD and age (RERI = -0.16 for the 75–84 age-group and RERI = -0.20 for the 85 + age-group). This means that on an additive scale having CVD is protective for the incident falls risk in the older age-groups compared to the youngest age group (65–74).

The inclusion of a new CVD diagnosis within the first 90 days post-index ED fall (Appendices 3 and 4) and the associated sensitivity analyses (Appendices 7 and 8) did not show any substantive change from the results reported for incident falls in Table 2 and Appendix 3.

### *Predictive power of clinical indicators to discriminate incident falls (*≥ *1 versus none) within the ED fall group*

Figure 2 presents ROCs predicting three-year incident falls for the ED fall group, stratified by 65–74, 75–84 and 85 + age groups, together with the AUC based on logistic regression models. The AUC ROC indicated that there was a 64% chance that the model could discriminate incident falls over three years. The AUC decreased with age indicating that the predictive power of the model to discriminate incident falls was very modest for the 65–74 age group but poor for the 85 + age group. Appendix 5 shows the same AUC ROC with CVD removed from the model, which did not change the power of the model to predict incident falls.

### All-cause mortality among fall versus non-fall groups of ED attendees

Appendix 6 presents the incidence percentage of three-year mortality for ED attendees aged ≥ 65 years with no fall, a fall, and a fall with hip fracture at the index ED admission. The data is presented for 65–74, 75–84 and 85 + age groups. Within each group of ED attendees, there was a progressive age-associated increase in the incident percentage of mortality. Within each age group, the incident percentage of mortality was highest for the ED fall with hip fracture group, ranging from 26.8 to 61.6%. Interestingly the incident percentage of mortality was lower among the ED fall compared to non-fall groups, but this difference was only significant within the 65–74 age group.

### Clinical risk indicators of all-cause mortality within the ED fall group

Table 2 presents prospective associations between clinical risk indicators at the index fall and mortality over 3 years. Age (75–84 years: adjusted RR = 2.32, and 85 + years: RR = 4.89), a history of hip fracture (RR = 1.31), a history of falls (1 fall: RR = 1.12, and > 1 fall: RR = 1.41), polypharmacy (5–10 medications: RR = 1.44, and > 10 medications: RR = 2.07) and a CVD diagnosis (RR = 1.81) were statistically significant predictors of 3 year mortality within the ED fall group. Taking CVD medications (RR = 0.95) reduced the risk of mortality in fully adjusted models.

Compared to the ED fall group aged 65–74 years without a CVD diagnosis, having CVD diagnosis and being aged 75–84 increased the adjusted RR of mortality over threefold, with a greater than fivefold increased risk for the ED fall group with a CVD diagnosis in the 85 + age group. Similarly, a CVD diagnosis and 1 pre-index fall doubled the risk of mortality in the ED fall group, while a CVD diagnosis and > 1 pre-index fall increased the risk of mortality 2.3-fold compared to having no CVD diagnosis with no pre-index falls.

To further investigate the second order interactions between CVD diagnosis and age and CVD diagnosis and pre-index fall history, we computed the RR per stratum, multiplicative interaction RR, and RERI to estimate the additive interaction (Appendix 4) [30]. The RR per stratum shows the relative risk of mortality within age groups and groups with different pre-index fall histories. Compared to the ED fall group aged 65–74 years, being aged 75–84 increased the adjusted RR of mortality by 43%, with a 9% increased risk in the 85 + age group. Compared to the ED fall group without a pre-index fall, having 1 pre-index fall increased the adjusted RR of mortality by 79%, with a 65% increased risk for > 1 pre-index fall. The multiplicative interaction effects between CVD, age and fall history show that the combined effect of having a CVD diagnosis and being older is less than the product of the individual effects of CVD and age (RR = 0.79 for the 75–84 group and RR = 0.60 for the 85 + age group). This means that having a CVD diagnosis has a protective effect on the mortality risk for older ED fallers on a multiplicative scale. The RERI or additive interaction effect shows that the combined effect of having a CVD diagnosis and being age 85 + is less than the sum of the individual effects of CVD diagnosis and age (RERI = -0.35). This means that on an additive scale having CVD diagnosis is only protective for mortality risk in the 85 + age group.

The inclusion of a new CVD diagnosis within the first 90 days post-index ED fall (Appendices 3 and 4) and the associated sensitivity analyses (Appendices 7 and 8) did not show any substantive change from the results reported for mortality in Table 2 and Appendix 3.

### Predictive power of clinical indicators to discriminate mortality over 3 years within the ED fall group.

Supplementary Appendix 9 presents ROCs predicting three-year all-cause mortality for the ED fall group stratified by 65–74, 75–84 and 85 + age groups, together with the AUC based on logistic regression models. The AUC ROC indicated that there was a 78% chance that the model could discriminate mortality over three years. The AUC decreased with age indicating that the predictive power of the model to discriminate mortality within the ED fall group was fair for the 65–74 age group but very modest for the 85 + age group.

## Discussion

In one of the largest studies of adults aged over 65 years attending the ED, utilising data from national administrative registers in Denmark, we have shown that the presence of a CVD was not a significant clinical predictor of incident falls over three years. Overall, the risk of incident falls was twofold higher among age and gender matched adults aged ≥ 65 years who had attended the ED for a fall versus another reason at baseline. The ED fall group, with and without hip fracture, are at greatest risk of incident falls over 3 years. Most notable was that older adults who attended the ED for a fall with a hip fracture at baseline, were at greatest risk for incident falls over 3 years in the younger 65–74 age group. While at all age groups they were also at the highest risk for mortality, increasing from 27 to 62% in the 65–74 and 85 + age groups respectively.

Conversely, the risk of mortality over three years was significantly higher among older adults who had attended the ED for another reason compared to those who attended for a fall. The higher percentage of hospitalisations following the ED attendance at baseline and the higher three-year mortality risk, would suggest that the ED non-fall group were more medically complex and potentially had more frailty than the age and gender matched fall group.

There was a significant prospective association between CVD diagnosis and mortality over three years among the ED fall group. However, a prior diagnosis of CVD was not a clinically significant factor in presenting with a fall at the ED, nor does it contribute significantly to the prediction of incident falls over three years, even if we also consider new CVD diagnosed within 90 days of the index ED presentation with a fall.

Previous studies have shown that most presentations to the ED with a fall are likely due to direct injury as the result of accidental falls, while a smaller percentage (10–15%) are due to syncope, faint or blackout with 30–40% of these due an underlying CVD e.g., hypotension or arrhythmia [31, 32]. The ED fall group also took significantly more CVD medications than the non-fall group and CVD medication use was associated with reduced risk of incident falls and mortality over 3 years. This is consistent with new emerging data that suggests that treating hypertension, the most common CVD, also has benefits in controlling orthostatic hypotension—a known risk factor for falls [33]. Other factors such as advancing age, history of pre-index falls, and polypharmacy were more clinically relevant when predicting incident falls among ED attendees with a fall at baseline. Indeed, a CVD diagnosis in combination with a prior history of falls doubled the risk of incident falls and mortality among those who attended the ED with a fall.

There are a number of plausible reasons why falls and cardiovascular disorders may be causally linked. Hypotension may cause cerebral hypoperfusion, resulting in syncope [34, 35]. If syncope is unwitnessed, which is the case in a majority of these events in older adults, the patient may have amnesia for loss of consciousness and thus be unaware of having fallen [36, 37]. Alternatively, compromised blood flow to neural pathways which govern gait and balance may induce instability and falls [21, 38, 39]. Cerebral white matter lesions are more common in patients with hypertension associated microvascular disease and further contribute to fall risk through dysregulated neural pathways governing gait, balance, mobility, cognitive and mood impairments [40–43]. Most cardiovascular diseases can be treated and represent potentially modifiable risk factors for falls if recognized and treated before irreparable damage occurs [44, 45]. Furthermore, polypharmacy, which was highest among the ED falls group, may cause or exacerbate orthostatic hypotension and impair cognitive functions such as concentration and executive function, thereby contributing to falls [46–48].

The power to predict incident falls using a small number of established clinical risk factors for falls, including CVDs, was modest at 0.64. This is generally consistent with other studies that model falls risk on a limited number of routinely available clinical indicators. More accurate falls prediction models often require a larger number of parameters including computational gait and balance parameters not routinely or feasibly collected in ED settings. [49–51].

Current World Falls Prevention Guidelines underline the significance of a cardiovascular evaluation in the assessment and management of falls risk [16]. However, research in this area is scarce and in current medical practice a comprehensive systematic cardiovascular assessment (CVD history and autonomic function testing) is not routinely performed as part of falls risk assessment and management [22, 23]. The findings in this study raise questions regarding if, when, and on whom, we should perform cardiovascular evaluations as part of a falls risk assessment [52]. Our data would suggest such evaluations may not be the highest priority when assessing older adults presenting to the ED with a fall. Randomized Control Trials (RCTs) are necessary to determine whether intervention for CVD, particularly in the those who attend ED for a fall with and without hip fracture, will reduce the risk of further falls and mortality.

Strengths of this study include the robust matched cohort study design and the large nationally representative dataset from administrative registers in Denmark. A further strength of this study is the capture of falls, clinical and medication data as part of the Danish National Patient Registry and the National Prescription Registry. This mitigates against the problem of recall bias inherent to many studies using retrospective falls data. Frailty, multimorbidity and diagnosis of cognitive impairment or dementia were not measured directly, which is a limitation of the study. However, these conditions are captured and represented to some extent by inclusion of the number of medications prescribed. We also acknowledge that survivor effects among the oldest older fallers may explain the attenuated impact of CVD on incident falls and mortality risk. Competing risk survival analyses for mortality and incident falls to exclude this possibility was beyond the scope of the study.

## Conclusion

Older adults attending the ED with a fall, including those with hip fracture, were at greatest risk for future falls. While CVD did not predict incident falls, it increased the risk of mortality in the three-year follow up. The data we have presented would suggest that when developing new services for ED, those who attend for a fall, and potentially for a fall with hip fracture, are less medically complex from a CVD perspective. This may be informative for the provision of care pathways for older adults attending the ED due to a fall.

### Supplementary Information

**Additional file 1: Appendix 1. **Graphical representation of the study design. **Appendix 2.** Median (inter-quartile range - IQR) follow-up times in days (1096 = 3 years) for the different age-groups. **Appendix 3.** Relative risk of incident falls (≥1 versus 0) and all-cause mortality associated with clinical indicators within the ED fall group, with pre-index ED fall CVD diagnosis only (Original) from Table 2, or pre- and post-index ED fall CVD diagnosis (New CVD). **Appendix 4.** Measures for assessing multiplicative and additive interaction effects in the risk of three-year incident falls (≥1 versus 0) and all-cause mortality models within the ED fall group, with pre-index ED fall CVD diagnosis only (‘Original’), or pre and post index ED fall CVD diagnosis (‘New CVD’). **Appendix 5.** Receiver operating curves predicting three-year incident falls for the ED fall group (left) and per age group (right) together with the AUC based on logistic regression models for incident falls, *without CVD predictors*. The dotted line indicates an AUC of 0.5. **Appendix 6.** The incidence percentage of three-year all cause mortality for fall, fall with hip fracture, and non-fall ED attendees by age. **Appendix 7**. Sensitivity analysis showing the ‘Original’ analysis from Table 2, excluding (n=105) individuals with three-year incident fall/mortality before a new CVD diagnosis within the first 90 days post-index ED fall (n=41,041), to reflect the same group as the ‘New CVD’ analysis. **Appendix 8**. Measures for assessing multiplicative and additive interaction effects in the risk of three-year incident falls (≥1 versus 0) and all-cause mortality models for the sensitivity analysis in Appendix 6. **Appendix 9. **Receiver operating curves predicting three-year all-cause mortality for the ED fall group (left) and per age group (right) together with the AUC based on logistic regression models for all-cause mortality. The dotted line indicated an AUC of 0.5.

## Data Availability

This study involved the secondary analyses of data from census based administrative registers in Denmark (https://www.dataforgood.science) under the central authority of Statistics Denmark (https://www.dst.dk/en#). Data may be accessed by application to Statistics Denmark.

## Abbreviations

ATC: Anatomical Therapeutic Chemical
AUC: Area Under the Curve
CVD: Cardiovascular disease
CI: Confidence Interval
DNPR: Danish National Patient Registry
ED: Emergency Department
EU: European Union
ICD-10: International Classification of Diseases-10
IQR: Interquartile range
RERI: Relative Excess Risk due to Interaction
ROC: Receiver Operating Curve
RR: Relative Risk
