# Supplementary material for: Cardiovascular disease and the risk of incident falls and mortality among adults aged ≥ 65 years presenting to the emergency department: a cohort study from national registry data in Denmark
Source: BMC Geriatr. 2024 Jan 24;24:93. doi: 10.1186/s12877-023-04618-2 (PMC10809657; doi:10.1186/s12877-023-04618-2)
Supplement: Supplementary file 1 — Additional file 1: Appendix 1. Graphical representation of the study design. Appendix 2. Median (inter-quartile range - IQR) follow-up times in days (1096 = 3 years) for the different age-groups. Appendix 3. Relative risk of incident falls (≥1 versus 0) and all-cause mortality associated with clinical indicators within the ED fall group, with pre-index ED fall CVD diagnosis only (Original) from Table 2, or pre- and post-index ED fall CVD diagnosis (New CVD). Appendix 4. Measures for assessing multiplicative and additive interaction effects in the risk of three-year incident falls (≥1 versus 0) and all-cause mortality models within the ED fall group, with pre-index ED fall CVD diagnosis only (‘Original’), or pre and post index ED fall CVD diagnosis (‘New CVD’). Appendix 5. Receiver operating curves predicting three-year incident falls for the ED fall group (left) and per age group (right) together with the AUC based on logistic regression models for incident falls, without CVD predictors. The dotted line indicates an AUC of 0.5. Appendix 6. The incidence percentage of three-year all cause mortality for fall, fall with hip fracture, and non-fall ED attendees by age. Appendix 7. Sensitivity analysis showing the ‘Original’ analysis from Table 2, excluding (n=105) individuals with three-year incident fall/mortality before a new CVD diagnosis within the first 90 days post-index ED fall (n=41,041), to reflect the same group as the ‘New CVD’ analysis. Appendix 8. Measures for assessing multiplicative and additive interaction effects in the risk of three-year incident falls (≥1 versus 0) and all-cause mortality models for the sensitivity analysis in Appendix 6. Appendix 9. Receiver operating curves predicting three-year all-cause mortality for the ED fall group (left) and per age group (right) together with the AUC based on logistic regression models for all-cause mortality. The dotted line indicated an AUC of 0.5. [file 12877_2023_4618_MOESM1_ESM.docx]

**The risk of incident falls and mortality among adults aged ≥65 years** **presenting to the Emergency Department: A cohort study from national registry data in Denmark.**

**Supplementary Maerial.**

**Appendix 1.** Graphical representation of the study design. 3

**Appendix 2.** Median (inter-quartile range - IQR) follow-up times in days (1096 = 3 years)

for the different age-groups. 4

**Appendix 3:** Relative risk of incident falls (≥1 versus 0) and all-cause mortality associated with clinical indicators within the ED fall group, with pre-index ED fall CVD diagnosis only (Original) from Table 2, or pre- and post-index ED fall CVD diagnosis (New CVD). 5

**Appendix 4.** Measures for assessing multiplicative and additive interaction effects in the risk of three-year incident falls (≥1 versus 0) and all-cause mortality models within the ED fall group, with pre-index ED fall CVD diagnosis only (‘Original’), or pre and post index ED fall CVD diagnosis (‘New CVD’). 6

**Appendix 5.** Receiver operating curves predicting three-year incident falls for the ED fall group (left) and per age group (right) together with the AUC based on logistic regression models for incident falls, *without CVD predictors*. The dotted line indicates an AUC of 0.5. Note that the logistic regression models for the ROCs include all second order interactions except those between polypharmacy and excessive polypharmacy and fall history (1 fall) and fall history (> 1 fall) due to singularity. 7

**Appendix 6.** The incidence percentage of three-year all cause mortality for fall, fall with hip fracture, and non-fall ED attendees by age. 8

**Appendix 7**. Sensitivity analysis showing the ‘Original’ analysis from Table 2, excluding (n=105) individuals with three-year incident fall/mortality before a new CVD diagnosis within the first 90 days post-index ED fall (n=41,041), to reflect the same group as the ‘New CVD’ analysis. 9

**Appendix 8**. Measures for assessing multiplicative and additive interaction effects in the risk of three-year incident falls (≥1 versus 0) and all-cause mortality models for the sensitivity analysis in Appendix 6. The ‘Original’ analysis from supplementary Appendix 3, excluding (n=105) individuals with an incident fall/mortality before a ‘New CVD’ diagnosis within the first 90 days post-index ED fall (n=41,041). 10

**Appendix 9.** Receiver operating curves predicting three-year all-cause mortality for the ED fall group (left) and per age group (right) together with the AUC based on logistic regression

models for all-cause mortality. The dotted line indicated an AUC of 0.5. Note that the

logistic regression models for the ROCs include all second order interactions except

those between polypharmacy and excessive polypharmacy and fall history (1 fall) and

fall history (> 1 fall) due to singularity. 11

Population aged ≥65 years in Denmark in 2013 n=1,070,428

Cohort aged ≥65 years in Denmark who attended the ED in 2013

n=135,637

Age and sex matched, attended ED for reason other than a fall in 2013

n=41,146

Attended the ED due to a fall in 2013

n=41,146

Age and sex matched, attended ED for reason other than a fall in 2013

with 3-year follow-up n=41,146

Attended the ED due to a fall in 2013 with 3-year follow-up

n=41,146

Attended the ED due to a fall in 2013 with 3-year follow-up, without a new fall/death before a new CVD diagnosis within 90 days of index ED falls visit.

(New CVD Unmasked)

n=41,041

**Appendix 1:** Graphical representation of the study design.

**Appendix 2.** Median (inter-quartile range - IQR) follow-up times in days (1096 = 3 years) for the different age-groups

| **Age-group**  **(years)** | **’Fallers’**  **ED attendees** | **‘Non-faller’**  **ED attendees** |
| --- | --- | --- |
| 65-74 | 1096 (1095, 1096) | 1096 (1095, 1096) |
| 75-84 | 1096 (908, 1096) | 1096 (652, 1096) |
| 85+ | 901 (277, 1096) | 838 (178, 1096) |

**Appendix 3:** Relative risk of incident falls (≥1 versus 0) and all-cause mortality associated with clinical indicators within the

ED fall group, with pre-index ED fall CVD diagnosis only (Original) from Table 2, or pre- and post-index ED fall CVD

diagnosis (New CVD).

|  | **New falls (≥1 versus none) ^1^** | | **All-cause mortality^1^** | |
| --- | --- | --- | --- | --- |
|  | Original  (n=41,146) | New CVD†  (n=41,041) | Original  (n=41,146) | New CVD†  (n=41,041) |
| Intercept | 1.00 | 1.00 | 1.00 | 1.00 |
| Age 75-84 | 1.32 (1.25; 1.40) * | 1.30 (1.23; 1.39) * | 2.32 (2.16; 2.49) * | 2.35 (2.17; 2.54) * |
| Age 85+ | 1.54 (1.45; 1.63) * | 1.57 (1.48; 1.67) * | 4.89 (4.58; 5.22) * | 5.15 (4.79; 5.54) * |
| Hip fracture | 0.88 (0.81; 0.95) * | 0.87 (0.80; 0.95) * | 1.31 (1.25; 1.38) * | 1.30 (1.24; 1.36) * |
| Fall history (1 fall)^2^ | 1.50 (1.42; 1.59) * | 1.54 (1.45; 1.64) * | 1.12 (1.06; 1.18) * | 1.16 (1.09; 1.23) * |
| Fall history (>1 fall)^2^ | 2.04 (1.89; 2.19) * | 2.03 (1.87; 2.20) * | 1.41 (1.31; 1.53) * | 1.51 (1.39; 1.64) * |
| Polypharmacy^2^ | 1.33 (1.24; 1.44) * | 1.33 (1.23; 1.44) * | 1.44 (1.32; 1.56) * | 1.42 (1.30; 1.54) * |
| Excessive polypharmacy^2^ | 1.51 (1.40; 1.62) * | 1.50 (1.39; 1.61) * | 2.07 (1.91; 2.24) * | 2.03 (1.87; 2.20) * |
| Cardiovascular medication use^2^ | 0.94 (0.89; 0.98) * | 0.92 (0.88; 0.97) * | 0.95 (0.91; 0.99) * | 0.93 (0.88; 0.97) * |
| Cardiovascular disease^3^ | 1.33 (1.24; 1.43) * | 1.38 (1.29; 1.47) * | 1.81 (1.67; 1.97) * | 2.02 (1.85; 2.20) * |
| Cardiovascular disease:Age 75-84 | 1.50 (1.41; 1.60) * | 1.57 (1.48; 1.68) * | 3.31 (3.05; 3.58) * | 3.68 (3.39; 4.00) * |
| Cardiovascular disease:Age 85+ | 1.67 (1.57; 1.77) * | 1.72 (1.62; 1.83) * | 5.35 (4.95; 5.77) * | 6.02 (5.55; 6.52) * |
| Cardiovascular disease:Fall history (1 fall) | 1.84 (1.70; 1.99) * | 1.90 (1.76; 2.05) * | 2.00 (1.80; 2.22) * | 2.19 (1.97; 2.43) * |
| Cardiovascular disease:Fall history (>1 fall) | 2.37 (2.17; 2.58) * | 2.48 (2.28; 2.71) * | 2.32 (2.06; 2.62) * | 2.53 (2.25; 2.86) * |
| ^1^ Relative risk (RR) with 95% confidence intervals (95% CI). The last four lines represent the RR of the combination of groups (having CVD and in a specific age group or having a specific fall history) compared to the reference 65-74 year olds without CVD or fall history. Note that these four lines do not represent a test of the interaction of the effects of CVD, age and fall history.  ^2^ Three years prior to the index fall.  ^3^ A diagnosed cardiovascular disease during the actual medical history (five years prior to the index fall).  ^*^ Significance level p < 0.05. | | | | |

^†^ We investigated whether new CVD diagnosed after the index fall impacted the association between CVD and incident falls risk. New CVD diagnoses was defined as a CVD diagnosis in the first 90 days after the ED index fall for those that did not have a CVD diagnosis in the 5-year pre-index fall period. There were 3048 in the ED fall group (4411 in ED non-fall group) with a new CVD diagnosis after the ED index-fall, of which 1698 (3241 in ED non-fall group) obtained their diagnosis within 90 days. To assess the impact of the new CVD diagnosis, the regression models were repeated including these new CVD diagnoses. We excluded 105 of the 1698 in the ED fall group (79 in ED non-fall group) from the analysis because they had an incident fall or died before the new CVD diagnosis. Results for these analyses. We found that effect sizes for the clinical risk indicators for incident falls and mortality were only improved very modestly by combining pre- and post- index fall diagnosis of CVD.

**Appendix 4.** Measures for assessing multiplicative and additive interaction effects in the risk of three-year incident falls (≥1 versus 0)

and all-cause mortality regressions within the ED fall group, with pre-index ED fall CVD diagnosis only (Original), or pre and

post index ED fall CVD diagnosis (New CVD).

|  | **New falls (≥1 versus none)** | | **All-cause mortality** | |
| --- | --- | --- | --- | --- |
|  | Original  (n=41,146) | New CVD†  (n=41,041) | Original  (n=41,146) | New CVD†  (n=41,041) |
| **Within strata RR^1^** |  |  |  |  |
| Cardiovascular disease (Age 75-84) | 1.13 (1.07; 1.20) * | 1.21 (1.13; 1.28) * | 1.43 (1.33; 1.52) * | 1.57 (1.46; 1.68) * |
| Cardiovascular disease (Age 85+) | 1.33 (1.24; 1.43) * | 1.10 (1.03; 1.17) * | 1.09 (1.03; 1.16) * | 1.17 (1.10; 1.24) * |
| Cardiovascular disease (Fall history 1 fall) | 1.23 (1.12; 1.34) * | 1.23 (1.13; 1.35) * | 1.79 (1.58; 2.02) * | 1.89 (1.67; 2.14) * |
| Cardiovascular disease (Fall history more than 1) | 1.16 (1.04; 1.29) * | 1.22 (1.09; 1.37) * | 1.65 (1.41; 1.92) * | 1.68 (1.43; 1.97) * |
| **Multiplicative scale**^2^ |  |  |  |  |
| Cardiovascular disease:Age 75-84 | 0.85 (0.78; 0.92) * | 0.88 (0.81; 0.95) * | 0.79 (0.70; 0.88) * | 0.78 (0.69; 0.87) * |
| Cardiovascular disease:Age 85+ | 0.81 (0.75; 0.88) * | 0.80 (0.73; 0.87) * | 0.60 (0.54; 0.67) * | 0.58 (0.52; 0.64) * |
| Cardiovascular disease:Fall history (1 fall) | 0.92 (0.85; 1.00) | 0.90 (0.83; 0.97) * | 0.99 (0.90; 1.08) | 0.94 (0.85; 1.03) |
| Cardiovascular disease:Fall history (more than 1) | 0.87 (0.79; 0.96) * | 0.89 (0.80; 0.99) * | 0.91 (0.79; 1.04) | 0.83 (0.72; 0.96) * |
| **RERI**^3^ |  |  |  |  |
| Cardiovascular disease:Age 75-84 | -0.16 (-0.27; -0.04) * | -0.11 (-0.22; 0.00) | 0.17 (-0.05; 0.39) | 0.32 (0.08; 0.55) * |
| Cardiovascular disease:Age 85+ | -0.20 (-0.32; -0.08) * | -0.23 (-0.35; -0.10) * | -0.35 (-0.66; -0.05) * | -0.16 (-0.50; 0.18) |
| Cardiovascular disease:Fall history (1 fall) | 0.01 (-0.12; 0.14) | -0.02 (-0.15; 0.11) | 0.07 (-0.08; 0.21) | 0.01 (-0.14; 0.17) |
| Cardiovascular disease:Fall history (more than 1) | -0.01 (-0.22; 0.21) | 0.08 (-0.15; 0.30) | 0.10 (-0.14; 0.34) | 0.01 (-0.26; 0.27) |

^1^ Relative within strata risk (RR) with 95% confidence intervals (95% CI).

^2^ Raw multiplicative scale interaction effects.

^3^ Relative Excess Risk due to Interaction (RERI) with 95% confidence intervals (95% CI).

^*^ Significance level p < 0.05.

^†^ We investigated whether new CVD diagnosed after the index fall impacted the association between CVD and incident falls risk. New CVD diagnoses was defined as a CVD diagnosis in the first 90 days after the ED index fall for those that did not have a CVD diagnosis in the 5-year pre-index fall period. There were 3048 in the ED fall group (4411 in ED non-fall group) with a new CVD diagnosis after the ED index-fall, of which 1698 (3241 in ED non-fall group) obtained their diagnosis within 90 days. To assess the impact of the new CVD diagnosis, the regression models were repeated including these new CVD diagnoses. We excluded 105 of the 1698 in the ED fall group (79 in ED non-fall group) from the analysis because they had an incident fall or died before the new CVD diagnosis. Results for these analyses are presented in Table 2 and Appendix 3.


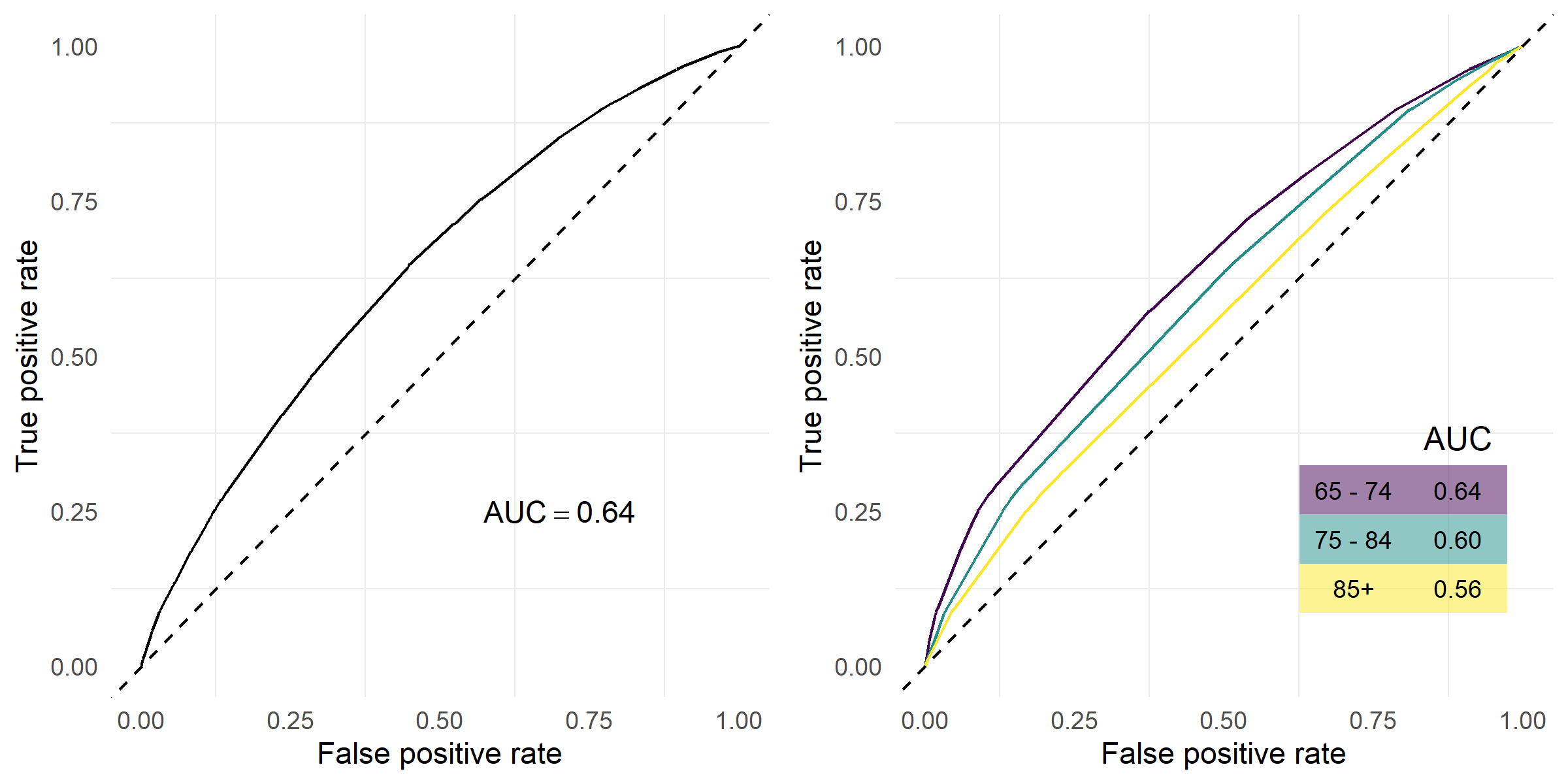


**Appendix 5.** Receiver operating curves predicting three-year incident falls for the ED fall group (left) and per age group (right)

Together with the AUC based on logistic regression models for incident falls, *without CVD predictors*. The dotted line indicates

an AUC of 0.5. Note that the logistic regression models for the ROCs include all second order interactions except those between polypharmacy and excessive polypharmacy and fall history (1 fall) and fall history (> 1 fall) due to singularity.


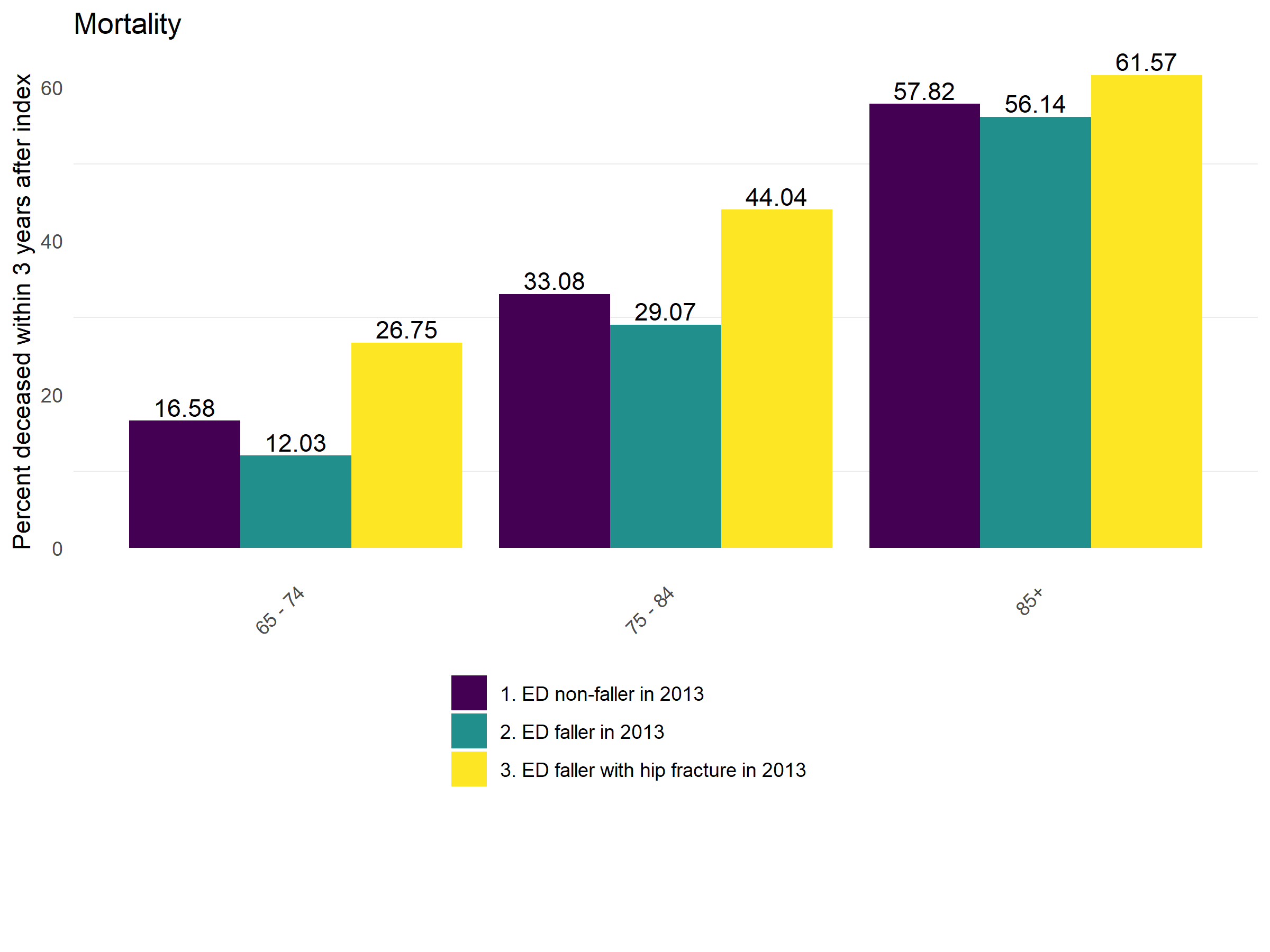


**Appendix 6.** The incidence percentage of three-year all cause mortality for fall, fall with hip fracture, and non-fall ED attendees by age.

**Appendix 7**. Sensitivity analysis showing the ‘Original’ analysis from Table 2, excluding (n=105) individuals with three-year

incident fall/mortality before a new CVD diagnosis within the first 90 days post-index ED fall (n=41,041), to reflect the

same group as the ‘New CVD’ analysis.

|  | **New falls**  **(≥1 versus none) ^1^** | **All-cause**  **mortality^1^** | |
| --- | --- | --- | --- |
|  | RR (95% CI) | RR (95% CI) | |
| Intercept | 1.00 | 1.00 | |
| Age 75-84 | 1.31 (1.24; 1.39) * | 2.32 (2.16; 2.49) * | |
| Age 85+ | 1.53 (1.45; 1.62) * | 4.89 (4.58; 5.23) * | |
| Hip fracture | 0.88 (0.81; 0.95) * | 1.31 (1.25; 1.38) * | |
| Fall history (1 fall)^2^ | 1.51 (1.43; 1.60) * | 1.12 (1.06; 1.18) * | |
| Fall history (more than 1)^2^ | 2.05 (1.90; 2.20) * | 1.42 (1.31; 1.53) * | |
| Polypharmacy^2^ | 1.33 (1.24; 1.44) * | 1.43 (1.31; 1.56) * | |
| Excessive polypharmacy^2^ | 1.51 (1.40; 1.62) * | 2.06 (1.90; 2.24) * | |
| Cardiovascular medication use^2^ | 0.93 (0.89; 0.98) * | 0.95 (0.91; 0.99) * | |
| Cardiovascular disease^3^ | 1.34 (1.25; 1.44) * | 1.82 (1.67; 1.98) * | |
| Cardiovascular disease:Age 75-84 | 1.51 (1.42; 1.61) * | 3.32 (3.07; 3.60) * | |
| Cardiovascular disease:Age 85+ | 1.68 (1.58; 1.79) * | 5.37 (4.97; 5.80) * | |
| Cardiovascular disease:Fall history (1 fall) | 1.86 (1.72; 2.01) * | 2.01 (1.81; 2.23) * | |
| Cardiovascular disease:Fall history (more than 1) | 2.39 (2.19; 2.61) * | 2.33 (2.07; 2.63) * | |
| ^1^ Relative risk (RR) with 95% confidence intervals (95% CI). The last four lines represent the RR of the particular groups (CVD and in a specific age group or having a specific fall history) compared to the reference 65-74 year olds without CVD or fall history. Note that these four lines do not represent a test of the interaction of the effects of CVD, age and fall history.  ^2^ Three years prior to the index fall.  ^3^ A diagnosed cardiovascular disease during the actual medical history (five years prior to the index fall).  * significance level p < 0.05. | | |  |

**Appendix** 8. Measures for assessing multiplicative and additive interaction effects in the risk of three-year incident falls (≥1 versus 0)

and all-cause mortality models for the sensitivity analysis in Appendix 6. The ‘Original’ analysis from supplementary Appendix 3, excluding (n=105) individuals with an incident fall/mortality before a ‘New CVD’ diagnosis within the first 90 days post-index ED

fall (n=41,041).

|  | **New falls**  **(≥1 versus none)** | **All-cause**  **mortality** |
| --- | --- | --- |
| **Within strata RR^1^** |  |  |
| Cardiovascular disease (Age 75-84) | 1.15 (1.08; 1.23) * | 1.43 (1.34; 1.53) * |
| Cardiovascular disease (Age 85+) | 1.10 (1.03; 1.17) * | 1.10 (1.04; 1.16) * |
| Cardiovascular disease (Fall history 1 fall) | 1.23 (1.12; 1.34) * | 1.79 (1.59; 2.02) * |
| Cardiovascular disease (Fall history more than 1) | 1.17 (1.04; 1.30) * | 1.65 (1.41; 1.93) * |
| **Multiplicative scale**^2^ |  |  |
| Cardiovascular disease:Age 75-84 | 0.86 (0.79; 0.93) * | 0.79 (0.71; 0.88) * |
| Cardiovascular disease:Age 85+ | 0.82 (0.75; 0.89) * | 0.60 (0.54; 0.67) * |
| Cardiovascular disease:Fall history (1 fall) | 0.91 (0.84; 0.99) * | 0.99 (0.90; 1.08) |
| Cardiovascular disease:Fall history (more than 1) | 0.87 (0.78; 0.96) * | 0.90 (0.79; 1.04) |
| **RERI**^3^ |  |  |
| Cardiovascular disease:Age 75-84 | -0.14 (-0.25; -0.03) * | 0.18 (-0.04; 0.40) |
| Cardiovascular disease:Age 85+ | -0.19 (-0.31; -0.07) * | -0.34 (-0.65; -0.03) * |
| Cardiovascular disease:Fall history (1 fall) | 0.00 (-0.13; 0.13) | 0.07 (-0.08; 0.21) |
| Cardiovascular disease:Fall history (more than 1) | -0.01 (-0.22; 0.21) | 0.10 (-0.15; 0.34) |

^1^ Relative within strata risk (RR) with 95% confidence intervals (95% CI).

^2^ Raw multiplicative scale interaction effects.

^3^ Relative Excess Risk due to Interaction (RERI) with 95% confidence intervals (95% CI).

* significance level p < 0.05.


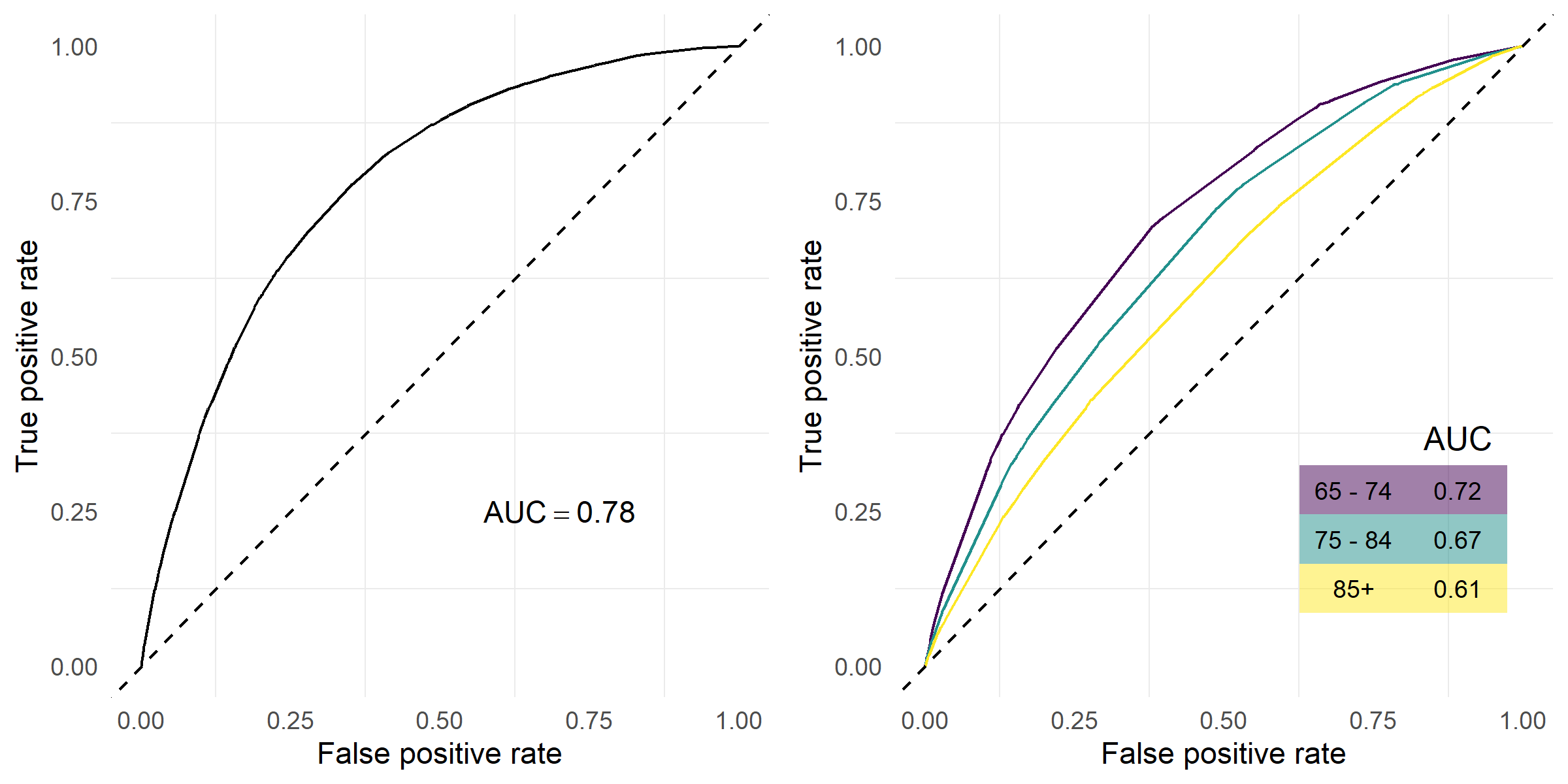


**Appendix 9:** Receiver operating curves predicting all-cause mortality for ED ‘fallers’ (left) and per age group (right) together with

the AUC based on logistic regression models for all-cause mortality. The dotted line indicated an AUC of 0.5. Note that the logistic regression models for the ROCs include all second order interactions except those between polypharmacy and excessive polypharmacy

and fall history (1 fall) and fall history (> 1 fall) due to singularity.
